# Immunohistochemical analyses of paraffin-embedded sections after primary surgery or trimodality treatment in esophageal carcinoma

**DOI:** 10.1016/j.ctro.2022.08.001

**Published:** 2022-08-03

**Authors:** Benjamin Terfa Igbo, Annett Linge, Susanne Frosch, Theresa Suckert, Liane Stolz-Kieslich, Steffen Löck, Mani Sankari Kumaravadivel, Thilo Welsch, Jürgen Weitz, Ulrich Sommer, Daniela Aust, Esther G.C. Troost

**Affiliations:** a Institute of Radiooncology - OncoRay, Helmholtz-Zentrum Dresden-Rossendorf, Rossendorf, Germany; b OncoRay - National Center for Radiation Research in Oncology, Faculty of Medicine and University Hospital Carl Gustav Carus, Technische Universität Dresden, Helmholtz-Zentrum Dresden – Rossendorf, Dresden, Germany; c Department of Radiotherapy and Radiation Oncology, Faculty of Medicine and University Hospital Carl Gustav Carus, Technische Universität Dresden, Dresden, Germany; d Department of Visceral, Thoracic and Vascular Surgery (VTG), Faculty of Medicine and University Hospital Carl Gustav Carus, Dresden, Germany; e Institute of Pathology, Faculty of Medicine and University Hospital Carl Gustav Carus, Technische Universität Dresden, Germany; f Institute for Pathology and Tumor and Normal Tissue Bank of the University Cancer Center (UCC), University Hospital Carl Gustav Carus, Medical Faculty, Technische Universität Dresden, Dresden, Germany; g German Cancer Consortium (DKTK), Partner Site Dresden, and German Cancer Research Center (DKFZ), Heidelberg, Germany; h National Center for Tumor Diseases (NCT), Partner Site Dresden, Germany; German Cancer Research Center (DKFZ), Heidelberg, Germany; Faculty of Medicine and University Hospital Carl Gustav Carus, Technische Universität Dresden, Dresden, Germany, and; Helmholtz Association / Helmholtz-Zentrum Dresden - Rossendorf (HZDR), Dresden, Germany.

**Keywords:** AC, Adenocarcinoma, AUC, Area under curve, BSA, Body surface area, CXCR4, Chemokine receptor type 4, CT, Computed tomography, CTV, Clinical target volume, FDG, [^18^F]-fluorodeoxyglucose, FFPE, Formalin-fixed paraffin-embedded, GTV, Gross tumor volume, HNSCC, Head and neck squamous cell carcinoma, HIF-1α, Hypoxia-inducible factor 1-alpha, IgG, Immunoglobulin, Ki67, Tumor proliferation nuclear protein, MRI, Magnetic resonance imaging, NRCHT +R, Neoadjuvant radiochemotherapy followed by resection, PD1, Programmed death 1 receptor, PET, Positron emission tomography, PTV, Planning target volume, p53, Tumor suppressor protein, RCHT, Radiochemotherapy, R, Resection, SCC, Squamous cell carcinoma, TME, Tumor microenvironment, UKD, University Hospital Carl Gustav Carus Dresden, 5-FU, 5-Fluorouracil, Tumor microenvironment, Esophageal cancer, Microscopic tumor extension, Radiochemotherapy, Whole slide image analysis

## Abstract

**Background:**

The microscopic tumor extension before, during or after radiochemotherapy (RCHT) and its correlation with the tumor microenvironment (TME) are presently unknown. This information is, however, crucial in the era of image-guided, adaptive high-precision photon or particle therapy.

**Materials and methods:**

In this pilot study, we analyzed formalin-fixed paraffin-embedded (FFPE) tumor resection specimen from patients with histologically confirmed squamous cell carcinoma (SCC; n = 10) or adenocarcinoma (A; n = 10) of the esophagus, having undergone neoadjuvant radiochemotherapy followed by resection (NRCHT + R) or resection (R)]. FFPE tissue sections were analyzed by immunohistochemistry regarding tumor hypoxia (HIF-1α), proliferation (Ki67), immune status (PD1), cancer cell stemness (CXCR4), and p53 mutation status. Marker expression in HIF-1α subvolumes was part of a sub-analysis. Statistical analyses were performed using one-sided Mann-Whitney tests and Bland-Altman analysis.

**Results:**

In both SCC and AC patients, the overall percentages of positive tumor cells among the five TME markers, namely HIF-1α, Ki67, p53, CXCR4 and PD1 after NRCHT were lower than in the R cohort. However, only PD1 in SCC and Ki67 in AC showed significant association (Ki67: p = 0.03, PD1: p = 0.02). In the sub-analysis of hypoxic subvolumes among the AC patients, the percentage of positive tumor cells within hypoxic regions were statistically significantly lower in the NRCHT than in the R cohort across all the markers except for PD1.

**Conclusion:**

In this pilot study, we showed changes in the TME induced by NRCHT in both SCC and AC. These findings will be correlated with microscopic tumor extension measurements in a subsequent cohort of patients.

## Highlights

• Changes in the tumor microenvironment of esophageal cancers, both in squamous cell carcinoma and adenocarcinoma, were found when comparing tumor resection specimen having undergone neoadjuvant radiochemotherapy followed by resection or resection only.
• Selected markers of the tumor microenvironment, i.e., Ki67, p53, CXCR4 and PD1 were found to be downregulated in hypoxic regions compared to normoxic regions.
• These findings will be correlated with microscopic tumor extension measurements in a subsequent, prospectively included cohort of esophageal cancer patients.

## 1 Introduction

The multimodality treatment of patients with esophageal cancer including radiochemotherapy (RCHT) followed by surgery is the cornerstone of their treatment [1], [2]. After neoadjuvant radiochemotherapy followed by resection (NRCHT + R), 32 % of patients develop a complete response, thus organ-preserving strategies are strived for [3]. Traditionally, radiotherapy has been delivered using photons, but there is increasing evidence that patients may indeed benefit from proton therapy, and a European study (PROTECT-TRIAL) comparing proton and photons irradiation in patients with esophageal squamous cell carcinoma (SCC) or adenocarcinoma (AC) is underway [4]. With increasingly used image-guided, adaptive techniques and treatment modalities with steeper dose gradients, more accurate and precise tumor demarcation is mandatory [5], [6]. This includes both the gross tumor volume (GTV) and the clinical target volume (CTV), the latter covering the GTV, and microscopic spread of the primary tumor. At present, the GTV prior to RCHT is derived from [^18^F]-fluorodeoxyglucose positron emission tomography (FDG-PET-imaging) and *endo*-esophageal endoscopy combined with ultrasound. However, these modalities fail to provide information about the microscopic extension of the primary tumor. During image-guided treatment adaptation using RCHT, information on cone-beam computed tomography (CT) or magnetic resonance imaging (MRI) is considered for the GTV, but again the CTV cannot be depicted.

It is hypothesized that the tumor microenvironment (TME), e.g. cancer stem cells, hypoxia, tumor cell proliferation, immune interaction, may influence the microscopic tumor extension and thus also the individual patients’ CTV margins, both prior to and during RCHT [7], [8], [9]. Data supporting this hypothesis are lacking to date. Esophageal cancer treated both with primary surgery or NRCHT + R depending on the tumor stage, is an ideal tumor entity to gather data for this. Histological specimens covering the tumor core as well as the oral and aboral parts of the esophagus are available for this analysis, thus representing the GTV and CTV.

Therefore, in order to prepare for a subsequent study allowing for a comprehensive assessment of the prospectively prepared resection specimens, i.e., using implantable markers illustrating the GTV, the first objective of this study was to compare changes in the TME in patients with esophageal adenocarcinoma (AC) or squamous cell carcinoma (SCC) treated with neoadjuvant radiochemotherapy followed by resection (NRCHT + R) with those in patients who underwent resection only (R). Secondly, an unbiased quantification tool for the assessment of the TME, abolishing the inter-observer variability, was established [10].

## 2 Materials and methods

### 2.1 Study cohort and ethical considerations

The study cohort consisted of 20 non-consecutive patients with esophageal cancer selected to contain four sub-cohorts of five patients each: Resection specimen of patients with esophageal cancer that received primary surgery (n = 10) or neoadjuvant radiochemotherapy followed by surgery (n = 10). Out of each subgroup, five patients had a histologically confirmed SCC or AC [i.e., five NRCHT + R and five R from each, SCC, and AC]. All patients were treated between 2014 and 2016 at the University Hospital Carl Gustav Carus Dresden (UKD), Germany. The Ethical Committee of the Technische Universität Dresden, Germany, approved the analysis on 26.09.2017 (EK 398102017). A written informed consent to use data for research purposes had previously been obtained from all patients. Tumor staging was done according to the Union for International Cancer Control (AJCC/UICC, 8th edition) [11]. Treatment decisions for all patients were taken in a multidisciplinary tumor board of the University Cancer Center: patients with < cT3N0M0 underwent R only and those with cT3 and/or cN + disease were treated according to the CROSS trial [12]. Two patients with loco-regionally advanced stage who were originally assigned to NRCHT + R underwent primary tumor resection, one due to age-related co-morbidity, the other for reasons of patient preference.

### 2.2 Patient characteristics and treatment regimen

All NRCHT + R patients underwent a diagnostic FDG-PET-CT scan within eight weeks prior to NCHRT, which also served for radiation treatment planning purposes. On the information obtained by FDG-PET-CT and *endo*-esophageal endoscopy, the GTV, CTV and planning target volume (PTV) were defined following local guidelines. Radiotherapy planning was performed using the Philips Pinnacle treatment planning system (version 9.8, Fitchburg, MA) applying intensity modulated radiation treatment technique. The NRCHT + R patients received a total dose of 40 Gy in 2 Gy fractions over the course of four weeks, except for two SCC patients who received 41.4 and 39.6 Gy, respectively, in 1.8 Gy fractions. Simultaneous chemotherapy was delivered with combinations of cisplatin and 5-fluorouracil (5-FU), or carboplatin and paclitaxel. All patients of the neoadjuvant treatment arm underwent surgery between five to seven weeks after the end of neoadjuvant therapy (Table1).

**Table 1:** Patient and treatment characteristics n = 20.

**Patient number**	**Tumor Type**	**Treatment**	**Gender**	**Age**	**Tumor stage (cT/cN)**	**RTx dose (Gy)**	**CTx agent**
1	SCC	R	M	67	cT3 cN0	none	none
2	SCC	R	M	48	cT2 cN0	none	none
3	SCC	R	M	62	cT1 cN0	none	none
4	SCC	R	F	81	cT2 cN1	none	none
5	SCC	R	F	45	cT2 cN0	none	none
6	SCC	NRCHT + R	M	53	cT3 cN1	40	cisplatin;5FU
7	SCC	NRCHT + R	M	63	cT4 cN1	40	cisplatin;5FU
8	SCC	NRCHT + R	F	60	cT3 cNX	40	cisplatin;5FU
9	SCC	NRCHT + R	M	57	cT3 cN2	41,4	carboplatin; paclitaxel
10	SCC	NRCHT + R	M	55	cT3 cN1	39,6	cisplatin;5FU
			**Mean (Range)**	59,1; (45–81)			
11	AC	R	F	80	cT3 cN+	none	none
12	AC	R	M	47	cT2 cN0	none	none
13	AC	R	M	64	cT1 cN0	none	none
14	AC	R	M	76	cT2 cN0	none	none
15	AC	R	M	62	cT1 cN0	none	none
16	AC	NRCHT + R	M	58	cT3 cN2	40	carboplatin; paclitaxel
17	AC	NRCHT + R	M	63	cT3 cN1	40	carboplatin; paclitaxel
18	AC	NRCHT + R	M	72	cT3 cN1	40	carboplatin; paclitaxel
19	AC	NRCHT + R	M	58	cT2 cN1	40	cisplatin;5FU
20	AC	NRCHT + R	M	51	cT3 cN1	40	cisplatin;5FU
			**Mean, (Range)**	63,1, (47–80)			

***Note*. SCC** = Squamous cell carcinoma, **AC** = Adenocarcinoma, **F** = Female, **M** = Male, **R** = Resection, **NRCHT + R** = Neoadjuvant radiochemotherapy followed by resection,

**5-FU** = 5-Fluorouracil.

### 2.3 Immunohistochemical staining

FFPE tumor tissue samples of patients with esophageal carcinoma were obtained from the Institute of Pathology [13]. For each patient, immunohistochemical staining and analyses of all the markers presented here was performed on two FFPE blocks of the primary tumor. Additional analyses of blocks obtained from the oral and aboral resection margin were unsuccessful, since these contained no (microscopic) tumor in the patients investigated. The FFPE tumor tissues were sectioned continuously into 3 µm-thick sections and further dewaxed in xylene for 3 × 10 min. H&E staining (Hematoxylin: Polyscience, Inc. Warrington, PA; Eosin: Sigma-Aldrich, St Louis, MO) for 40 and 30 s respectively was performed to confirm histological diagnosis. For immunohistochemical staining, rehydration was done by washing the sections in graded ethanol solutions, 2 × 100 %, 96 %, 80 %, 70 %, 40 % and PBS for 2 min each. The antigens were retrieved by heating the tissue sections in citrate buffer (pH 6) for 28 min in a microwave at 630 Watt. Afterwards, sections were cooled down on ice for 20 min. For immunohistochemical staining, blocking was done using peroxidase-block for 10 min. Thereafter sections were stained at room temperature for 30 min with monoclonal anti-human antibodies HIF-1α, (NB100-105: pH 6 1:20 dilution; Novus Biological, Centennial, CO), Ki67 (MIB-1: GA626, pH 6, 1:1500 dilution; Dako, Glostrup, Denmark), p53 (M7001: pH 9, 1:300, dilution; Dako), PD1 (NAT105: ab52587, pH 6, 1:50, dilution; Abcam, Cambridge, UK) and CXCR4 (ab124824: pH 6, 1:500; Abcam). The secondary antibody within the Envision-Kit (K5007: Dako) was incubated for 30 min at room temperature. Detection of antibody-binding was done by staining the sections with DAB for 10 min at room temperature followed by rinsing them in distilled water, thereafter counterstaining with hematoxylin solution (SAV 10231: Flinsbach a. Inn, Germany). Frequent washing steps with washing buffer (S3006: Dako) for 3 × 5 min were performed between consecutive steps. Slides were finally dehydrated and mounted in Entellan. Negative controls were processed similarly, and the corresponding host immunoglobulin (IgG) was applied.

### 2.4 Image acquisition and analysis

**Fig. 1:**
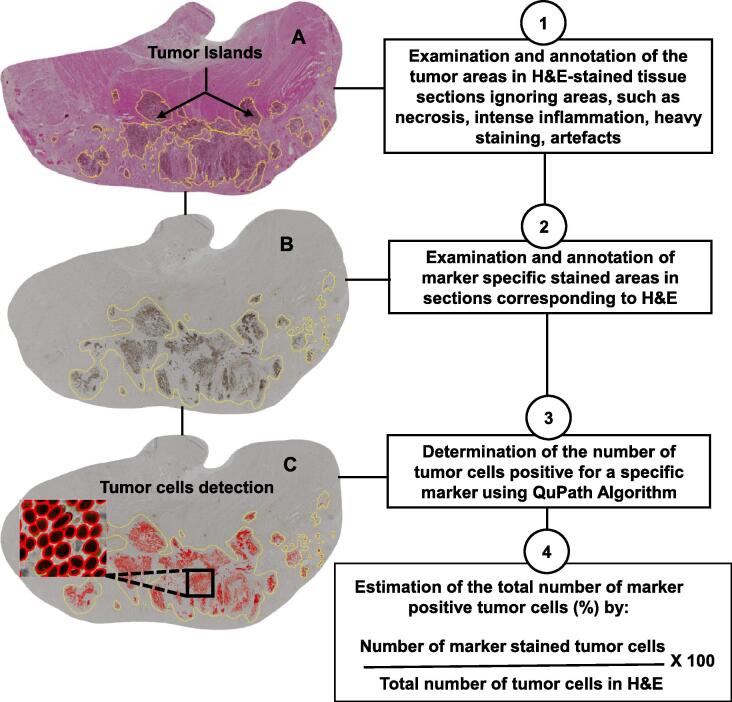
Workflow for assessment of the percentage of tumor cells positive for a specific marker. (A) H&E-stained tissue sections showing annotated tumor areas (tumor islands) in yellow mask. (B) Marker-specific stained tumor areas corresponding to H&E sections. (C) Detection of tumor cells positive for a specific marker using QuPath algorithm.

Microscopy imaging was performed on a Zeiss AxioScan.Z1 (Carl Zeiss AG, Feldbach, Switzerland), an automated slide scanner of the Light Microscopy Facility at the Core Facility of the CMCB Technology Platform at Technische Universität Dresden. Brightfield images were taken with a Zeiss Plan-Apochromat 10x/0.45 M27 objective and the color CCD camera, Hitachi HV-F202SCL (Akihabara UDX, Tokyo, Japan), with 4.4 µm pixel size, 24 bit and with uniform white balance. All tumor sections were analyzed in QuPath (version 0.2.3 University of Edinburg, UK) based on a computerized digital image-processing system using the segmentation method StarDist [14], [15]. After whole slide scan, an entire image was selected for analysis and imported into QuPath. The StarDist model was used for estimating positive tumor cells within the annotations. Positive tumor cells classifiers were trained, and quantification was based on the nuclear (Ki67, p53, HIF-1α, H&E and CXCR4) or membrane (PD1) staining specificity of each marker. PD1 was neither exclusively stained within the tumor cells nor TILS but rather within the entire tissue section. Before the implementation of the classifier-trained algorithm, the annotations of tumor regions within each section were manually outlined using the polygon tool for all the images. An experienced clinician (AL) validated these tumor annotations. Areas such as tumor necrosis and image artefacts were excluded from analyses. Data were extracted from QuPath, the ratio including percentages for each marker were further calculated in MS Excel by dividing the total number of positive tumor cells per each marker by the total number of tumor cells in the corresponding H&E section. The workflow of the QuPath image analysis is summarized in Fig. 1.

### 2.5 Estimation of tumor cells within and outside of hypoxia region

To calculate the co-localization of hypoxic tumor subvolumes and tumor characteristics within and outside those hypoxic subvolumes, annotations from hypoxic areas were masked onto the corresponding annotations of the other markers (Ki67, p53, CXCR4 and PD1). For this, the tumor was divided into two different regions: outer margin (marker expression outside of hypoxic area) and inner margin (marker expression within hypoxic area; see Fig. S1).

### 2.6 Concordance between QuPath and manual quantification

Manual tumor cell count is still considered the gold standard for the assessment of positive tumor cells. To confirm the accuracy of QuPath, the proportion of positively stained cells was manually counted by two independent observers (BI, TS) from 40 randomly selected stained tumor sections evenly distributed among the markers.

### 2.7 Statistical analysis

The analyses presented here were conducted on the average percentages of the two tumoral and intratumoral specimen of each patient. All the graphs and statistical analyses were performed using GraphPad Prism software version 8.0 for Windows (GraphPad Software, San Diego, CA). Since we expected that TME markers will be downregulated after NRCHT + R and in normoxic regions, we applied one sided Mann-Whitney tests to assess parameter differences between patient groups, and a p-value < 0.05 was considered significant. For verification of image analysis, interobserver variability and QuPath accuracy was performed using Bland-Altman algorithm with limits of agreement (bias ± 1.96 standard deviation).

## 3 Results

The Bland-Altman analysis showed a strong agreement between the average manual quantification from the two observers and the QuPath algorithm (mean difference −0.4125, SD ± 1.96) (Fig. S2). Therefore, only the automatically retrieved numbers are presented from hereon.

**Fig. 2:**
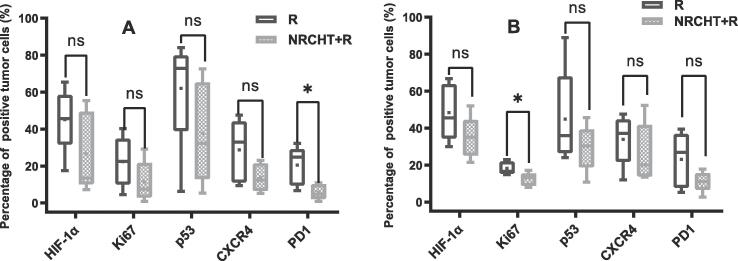
Percentage of positive tumor cells for (A) squamous cell carcinoma and (B) adenocarcinoma. * p < 0.05, ** p < 0.01, *** p < 0.001, ^ns^ not significant Mann-Whitney test.

In tumor resection specimen of both SCC and AC patients (Fig. 2A and B), the overall percentages of Ki67, p53, CXCR4 and PD1 positive tumor cells were lower in the NRCHT + R than in the R cohort. However, only for PD1 in SCC and Ki67 in AC this difference was statistically significant (Ki67: p = 0.03, PD1: p = 0.02) respectively.

**Fig. 3:**
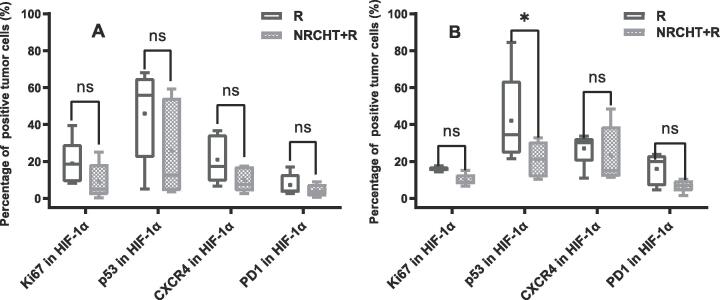
Percentage of positive tumor cells depending on hypoxia (HIF-1α) for (A) squamous cell carcinoma and (B) adenocarcinoma. * p < 0.05, ** p < 0.01, *** p < 0.001, ^ns^ not significant Mann-Whitney test.

Similarly, the expression of markers only within the hypoxic region (Fig. 3A and B) in both SCC and AC patients showed that the percentage of positive tumor cells in NRCHT + R was lower compared to R cohort, even though the difference was only significant for p53 in the AC cohort (p = 0.04).

**Fig. 4:**
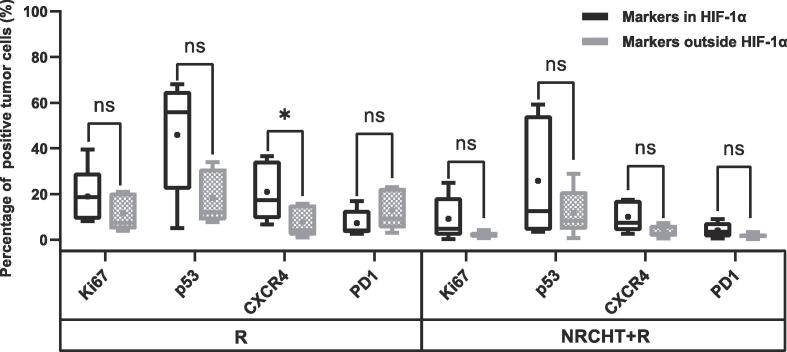
Percentage of positive tumor cells depending on hypoxia (HIF-1α) for (A) squamous cell carcinoma and (B) adenocarcinoma. * p < 0.05, ** p < 0.01, *** p < 0.001, ^ns^ not significant Mann-Whitney test.

**Fig. 5:**
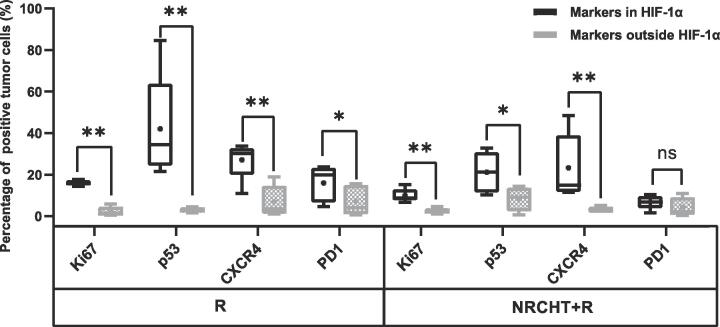
Percentage of positive tumor cells depending on hypoxia (HIF-1α) for (A) squamous cell carcinoma and (B) adenocarcinoma. * p < 0.05, ** p < 0.01, *** p < 0.001, ^ns^ not significant Mann-Whitney test.

In the sub-analysis of hypoxic subvolumes of AC patients (Fig. 4B), the percentage of Ki67, p53, CXCR4 and PD1 positive tumor cells were significantly higher within hypoxic regions compared to the normoxic regions regardless of the previous treatment, except for PD1 in the NRCHT + R cohort. Furthermore, the percentage of positive tumor cells across all the markers was higher within hypoxic regions compared to normoxic regions in SCC patients (Fig. 4A), but only CXCR4 in the R cohort was statistically significant (p = 0.04) (see Fig. 5).

## 4 Discussion

The results of this pilot study showed changes in the tumor microenvironment induced by NRCHT in both SCC and AC when compared to patients undergoing primary tumor resection. This was the case in the entire specimen, but also in subvolumes with HIF-1α positivity. Moreover, our results showed downregulation of the selected TME markers, i.e., HIF-1α, Ki67, p53, CXCR4 and PD1, within patients treated with NRCHT compared to patients receiving surgery only.

Some of our findings are in line with previous publications, while others differ. We found overexpression of CXCR4 after NRCHT + R compared to R alone in both esophageal SCC and AC patients. Koishi *et al.* [16] reported that persistent expression of CXCR4 correlates with distance recurrence and a worse overall survival in patients with esophageal cancer after RCHT. Data on the role of CXCR4 expression in esophageal cancer progression, and the prognosis of patients after RCHT are presently limited. So, it is to be confirmed whether CXCR4 signaling is a tumor microenvironmental factor inducing radiotherapy resistance. Recently published studies investigating PD1 and PDL1 following neoadjuvant RCHT of esophageal AC, revealed that PD1 expression was a better prognostic marker than PDL1 expression in AC [17]. In addition, the authors reported that higher expression of PD1 was associated with a significantly worst outcome. In contrast to this, Chen *et al*. [18] suggested that PDL1 could be a favorable indicator of prognosis in esophageal SCC. They found no significant correlation between PD1 expression and clinicopathological factors or outcome in esophageal SCC patients. However, this study was conducted in patients who underwent resection only. We observed that PD1 in both SCC and AC was more expressed in R than in NRCHT + R cohort. This observation and ideally the association of PDL1 expression is to be investigated in our subsequent, prospective study. Even though our results were only obtained in a small pilot study cohort, they are comparable to a previous study that used multiplex immunohistochemistry to predict TME response in esophageal carcinoma patients after multimodality treatment [19]. In that study, high expression of immune cells and infiltrating macrophages in TME positively correlated to poor treatment outcome and poor overall survival of patients with esophageal cancer. Therefore, our future analyses will also investigate the role of immune cells within the TME after RCHT using multiplex immune profiling approach [20].

Previous studies have shown that HIF-1α upregulation following NRCHT is associated with tumor cell proliferation, stemness and reduced immune response in esophageal and head and neck squamous cell carcinomas [21], [22]. Both studies demonstrated that high expression of HIF-1α, p53 and cancer stems cell marker were significantly associated with tumor recurrence, poor treatment outcome, and poor overall survival in patients with HNSCC treated with RCHT. Our present study showed that PD1 expression in AC was increased under hypoxic conditions compared to under normoxia following NRCHT + R. Similar results have been recently published [23]. Chen *et al*. [24] reported that HIF-1α upregulation correlated with increased PD1/PDL1 expression. They further found that HIF-1α expression levels positively correlated with the expression levels of tumor proliferation marker Ki67. This may underline the negative effect of hypoxia on treatment outcome.

Tumor hypoxia is a well-known microenvironmental parameter that regulates many biological processes leading to radiosensitivity, chemosensitivity, tumor progression and metastasis [25], [26]. Not surprising, hypoxic tumor subvolumes have been correlated with tumor evasion signatures such as tumoral immune escape, proliferation, mutational status and stemness [23], [24], [27]. In general, cancer stem cells represent a tumor subpopulation responsible for tumor metastasis and resistance to radiotherapy, ultimately leading to tumor relapse [28], [29], [30].

Whether the findings on altered TME are associated with changes in the microscopic tumor extension is to be assessed in the larger future cohort.

Our work contains several limitations apart from the small sample size. The samples were retrospectively retrieved from FFPE blocks, thus exact information of their *in vivo* localization in the patients was not available. Therefore, the correlation of markers of the TME with the microscopic tumor extension was not feasible in this cohort. Also, the radiation dose distribution in the NRCHT cohort could thus not be superimposed onto the blocks. Thirdly, we used consecutive tumor sections for the analysis and were not able to perform advanced multiplex staining at the time. Fourthly, the scanned tumor sections were manually aligned using QuPath, which holds the possibility of misalignment. Moreover, results on PDL1 staining, which was actually performed, were not included in these analyses, since the staining’s quality was suboptimal, whereas PD1 staining was of excellent quality and thus included in the analysis. Finally, patients who underwent NRCHT + R had more advanced tumor stage compared to those having undergone primary resection. This difference in tumor stage may have influenced the presented analyses, for which these need to be interpreted with some caution.

Thus, in the subsequent prospective cohort, fiducial markers will be placed on the borders of the tumors using endoscopic ultrasound guidance prior to imaging (planning CT and ideally FDG-PET-CT) and subsequent NRCHT. Moreover, a multiplex immunofluorescence staining protocol on biomarkers of TME associated with invasion and metastasis as well as different immune cells is currently being established. By doing so, we expect to unravel the correlation between these biomarkers of the tumor microenvironment and the microscopic tumor extension to improve clinical target volume definition.

## 6 Conclusion

This study showed changes in the tumor microenvironment induced by NRCHT in patients with SCC and AC of the esophagus. In particular, sub-analyses in hypoxic regions revealed changes compared to normoxic regions. QuPath provides an accurate and reproducible quantification method of positive tumor cells in whole tissue resection specimens stained with diverse markers. A larger study is planned to correlate immunohistochemical markers to the microscopic tumor extension.

AL and ET: Dr. Linge and Prof. Troost are involved in an ongoing publicly funded (German Federal Ministry of Education and Research) project with the companies Medipan (2019–2022), Attomol GmbH (2019–2022), GA Generic Assays GmbH (2019–2022), *Gesellschaft für medizinische und wissenschaftliche genetische Analysen* (2019–2022), Lipotype GmbH (2019–2022) and PolyAn GmbH (2019–2022). For the present manuscript, Dr. Linge and Prof. Troost confirm that none of the above-mentioned funding sources were involved.

## Declaration of Competing Interest

The authors declare that they have no known competing financial interests or personal relationships that could have appeared to influence the work reported in this paper.

## Appendix A Supplementary data

Supplementary data 1 Workflow for the determination of proliferation outside and inside of the hypoxic subvolume (HIF-1α) using the QuPath Distance to annotation command. (A) The annotation (yellow) of HIF-1α region. (B) Detections of Ki67 annotation (green) corresponding to HIF-1α tissue section to assess proliferation. (C) Masking of HIF-1α annotation (yellow) on Ki67 detected annotation (green): outer margin, magenta (Ki67 fractions outside hypoxic subvolumes) and inner margin, yellow (Ki67 hypoxic subvolumes).

Supplementary data 2 Bland-Altman analysis plots compare the number of positive tumor cells for (A) The manual count of the two independent observers. (B) The average count of the observers to the QuPath algorithm. Both counting methods had a comparable standard deviation with no general mean difference towards higher or lower positive tumor cell numbers (bias ± 1.96 standard deviation).

Supplementary data 3 QuPath quantification of H&E, p53 and Ki67: positive tumor cells in red annotations.

Supplementary data 4 QuPath quantification of HIF-1α, CXCR4 and PD1: positive tumor cells in red annotations.
