# Supplementary figures and images for: Immunohistochemical analyses of paraffin-embedded sections after primary surgery or trimodality treatment in esophageal carcinoma
Source: Clin Transl Radiat Oncol. 2022 Aug 3;36:106–12. doi: 10.1016/j.ctro.2022.08.001 (PMC9385880; doi:10.1016/j.ctro.2022.08.001)

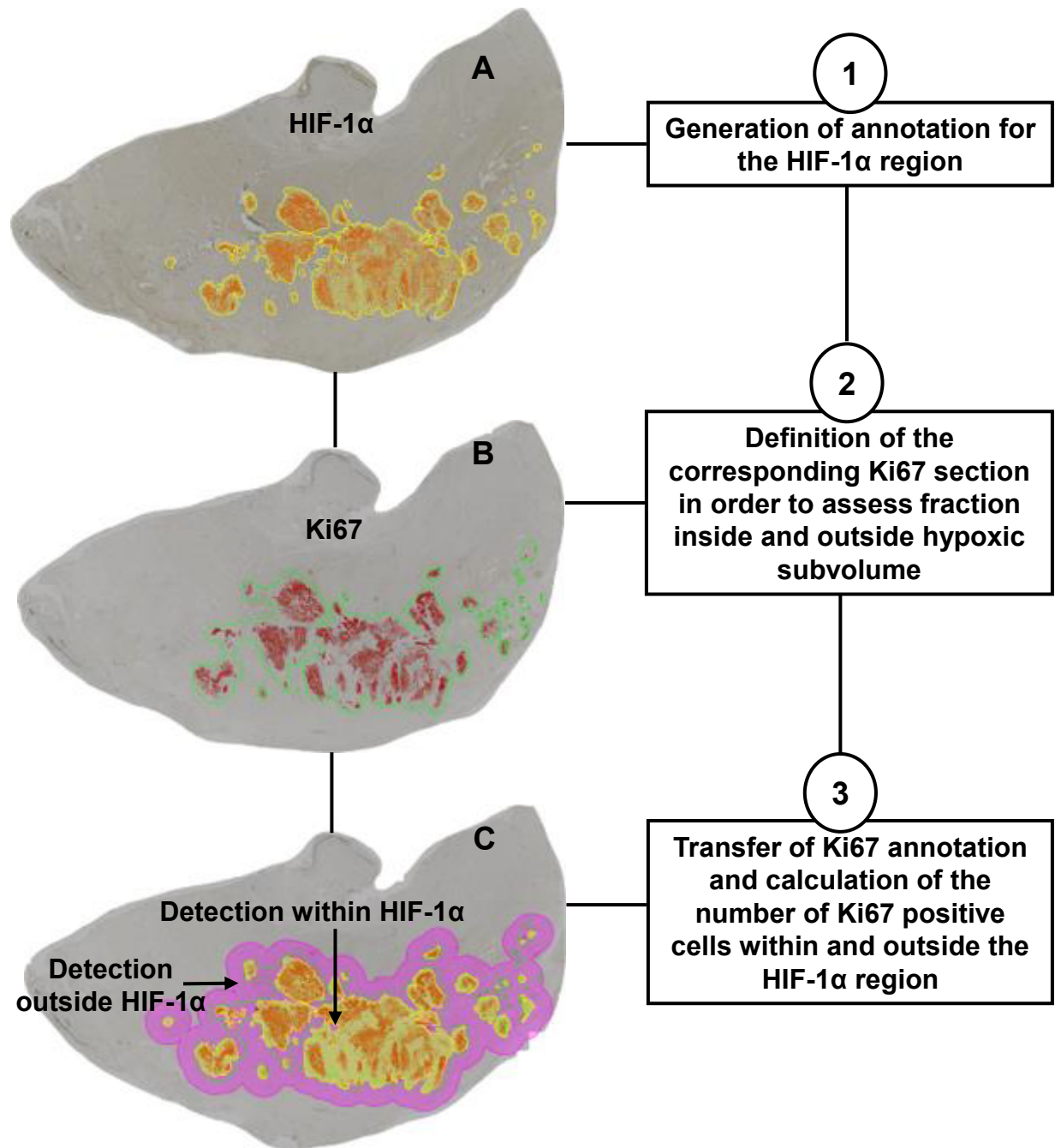

Supplement: Supplementary data 1 — Workflow for the determination of proliferation outside and inside of the hypoxic subvolume (HIF-1α) using the QuPath Distance to annotation command. (A) The annotation (yellow) of HIF-1α region. (B) Detections of Ki67 annotation (green) corresponding to HIF-1α tissue section to assess proliferation. (C) Masking of HIF-1α annotation (yellow) on Ki67 detected annotation (green): outer margin, magenta (Ki67 fractions outside hypoxic subvolumes) and inner margin, yellow (Ki67 hypoxic subvolumes). [file mmc1.pdf]

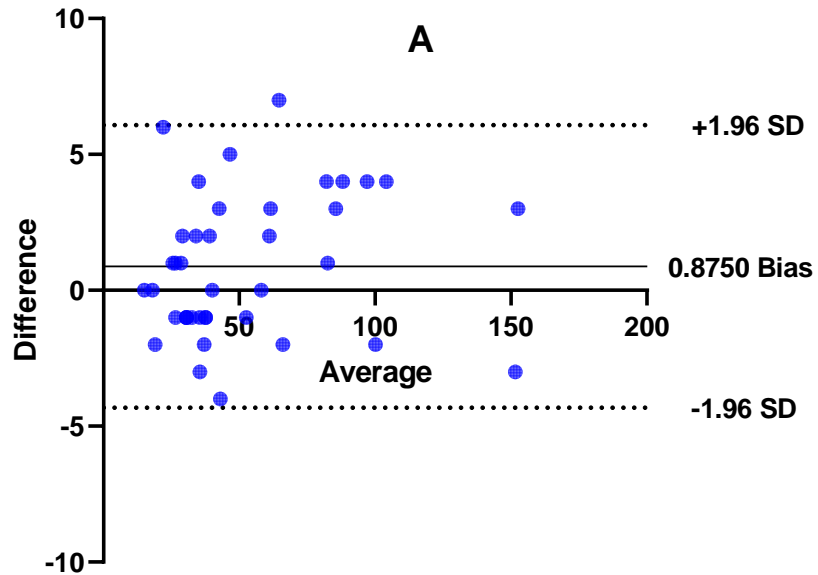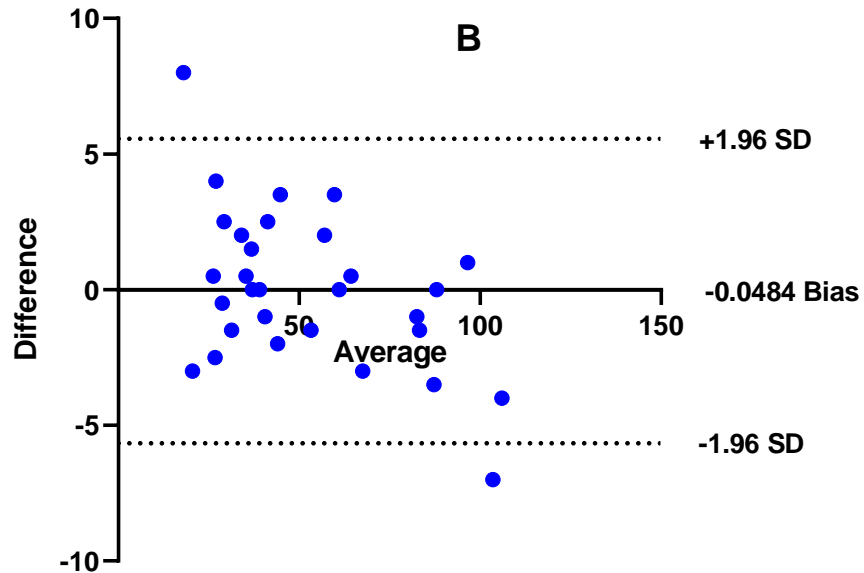

Supplement: Supplementary data 2 — Bland-Altman analysis plots compare the number of positive tumor cells for (A) The manual count of the two independent observers. (B) The average count of the observers to the QuPath algorithm. Both counting methods had a comparable standard deviation with no general mean difference towards higher or lower positive tumor cell numbers (bias ± 1.96 standard deviation). [file mmc2.pdf]

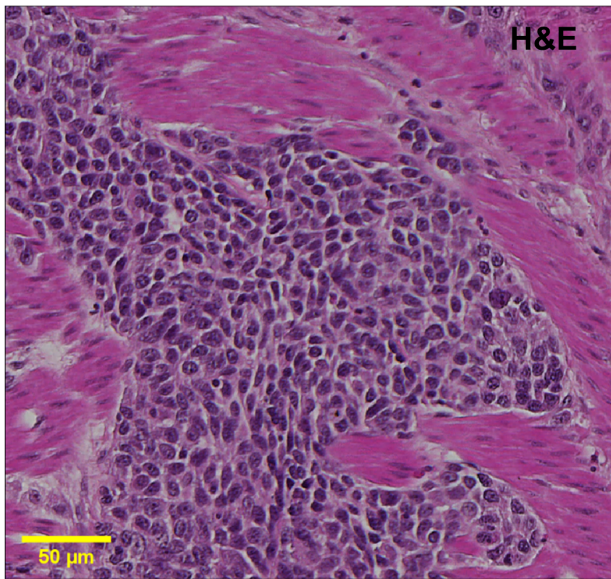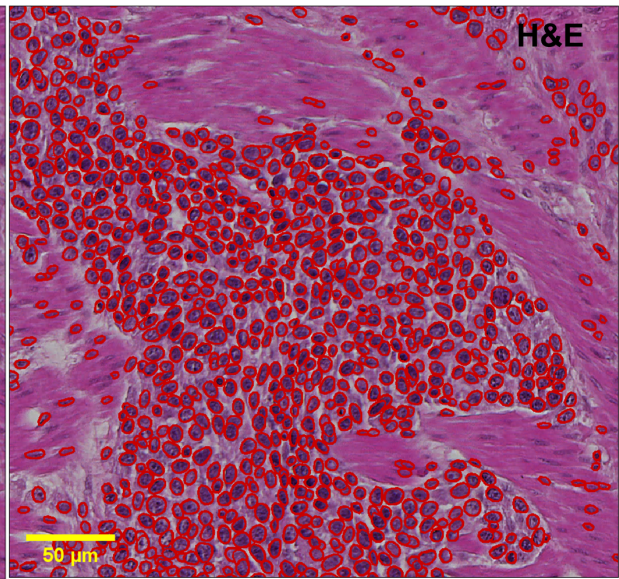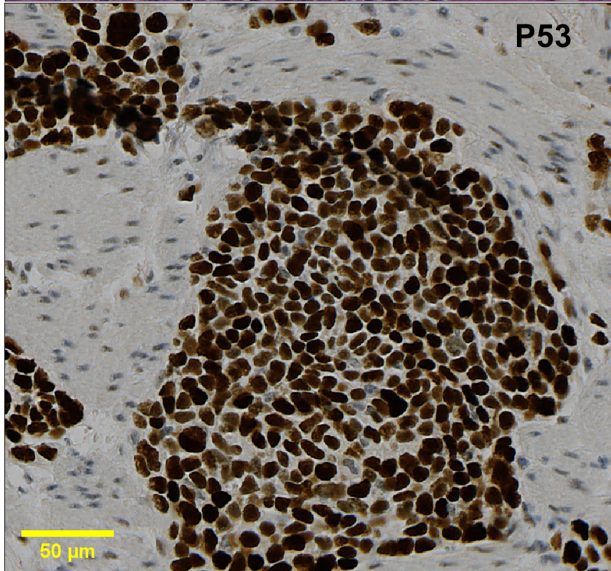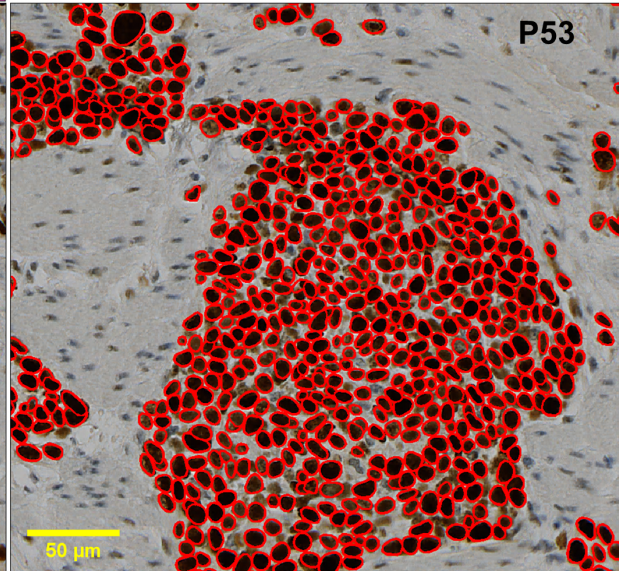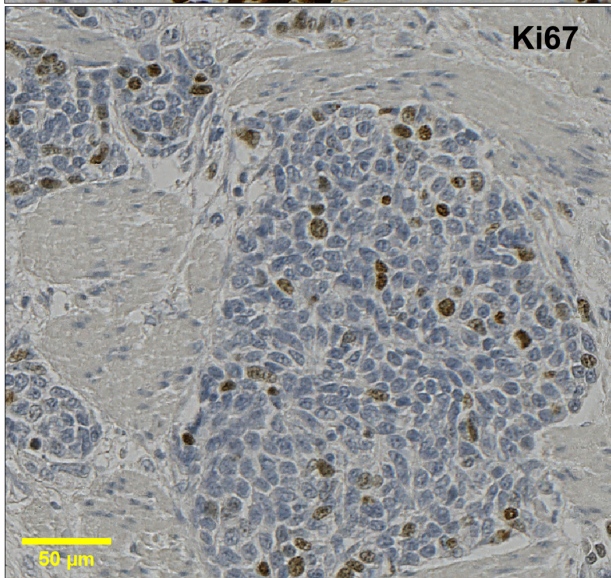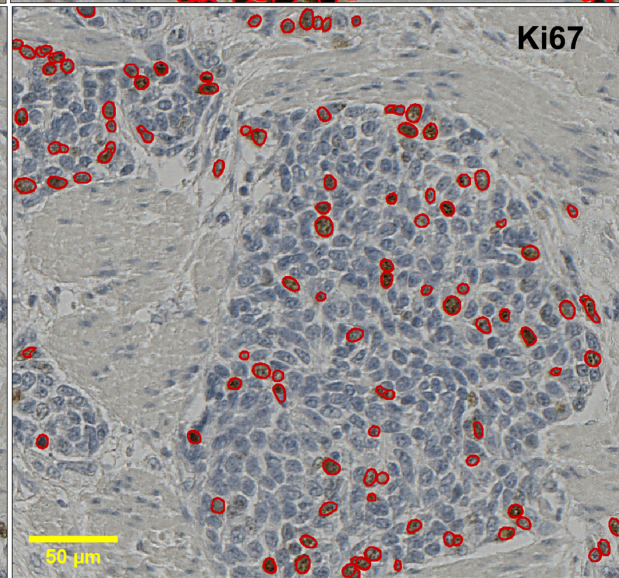

Supplement: Supplementary data 3 — QuPath quantification of H&E, p53 and Ki67: positive tumor cells in red annotations. [file mmc3.pdf]

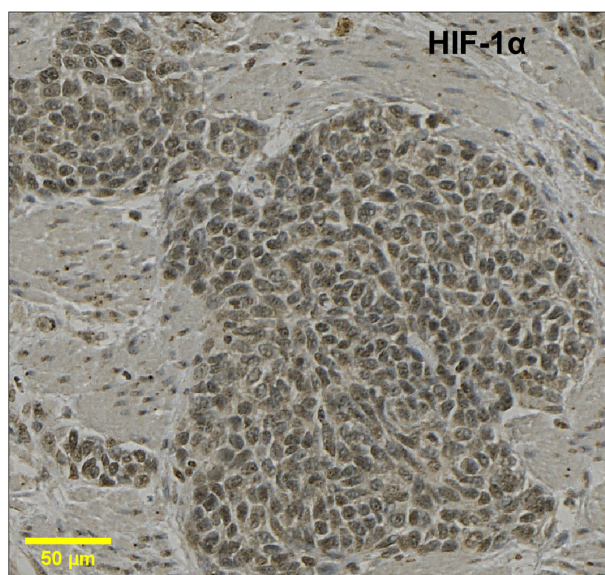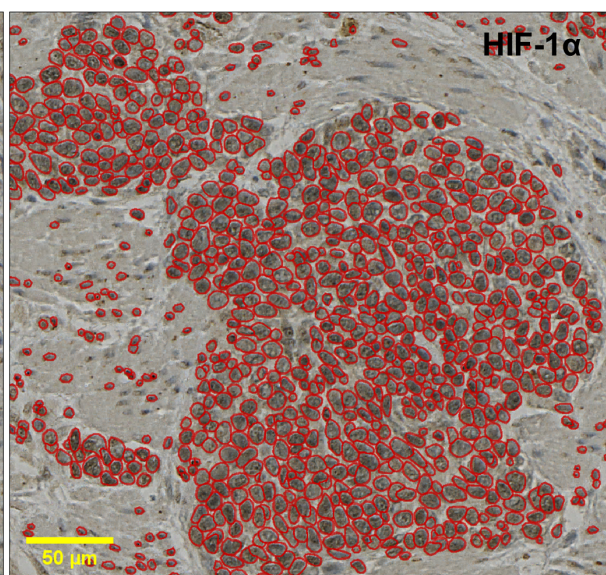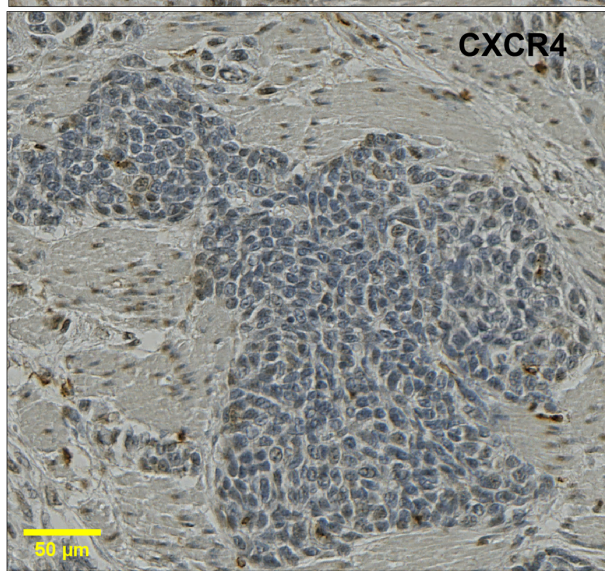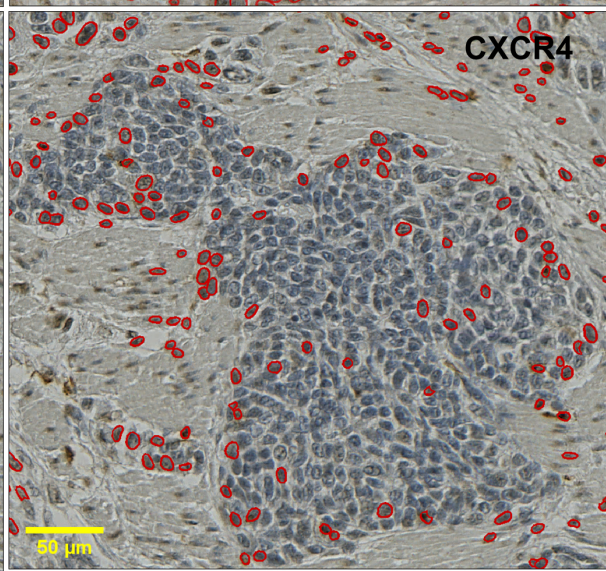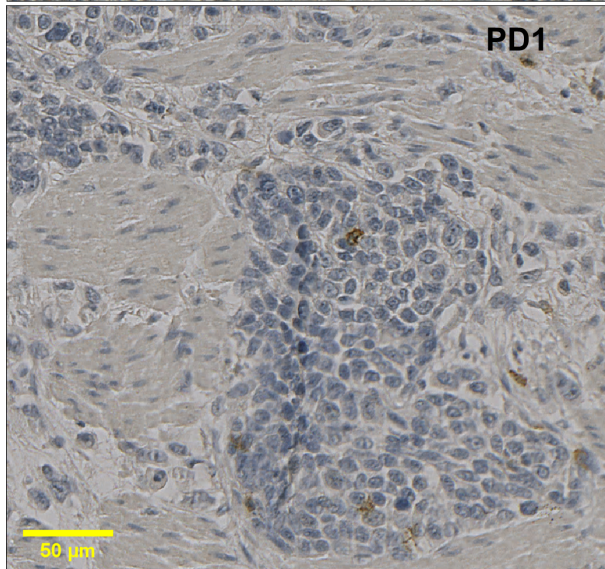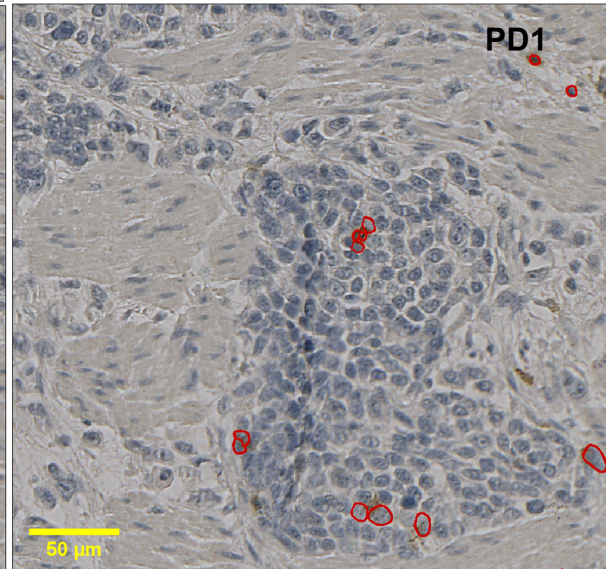

Supplement: Supplementary data 4 — QuPath quantification of HIF-1α, CXCR4 and PD1: positive tumor cells in red annotations. [file mmc4.pdf]
